# A comparison of the changes in serum lactate between surgical repair and transthoracic device closure of ventricular septal defects in pediatric patients

**DOI:** 10.3389/fcvm.2022.961997

**Published:** 2023-01-05

**Authors:** Jiang-Shan Huang, Yu-Kun Chen, Shi-Hao Lin, Qiang Chen, Hua Cao, Yi-Rong Zheng

**Affiliations:** Department of Cardiac Surgery, Fujian Children's Hospital (Fujian Branch of Shanghai Children's Medical Center), College of Clinical Medicine for Obstetrics and Gynecology and Pediatrics, Fujian Medical University, Fuzhou, China

**Keywords:** serum lactate, transthoracic device closure, surgical repair, ventricular septal defect, pediatric patients

## Abstract

**Objective:**

The purpose of this study was to compare the changes in serum lactate between surgical repair and transthoracic device closure of ventricular septal defects (VSDs) in pediatric patients.

**Methods:**

This study was a retrospective analysis, and 314 pediatric patients with simple VSD from October 2019 to October 2021 were selected. The patients were divided into the S group (surgical repair) and the D group (transthoracic device closure). The serum lactate value at ICU admission and 6 h after operation, as well as the highest serum lactate value were collected, and the 6-h serum lactate clearance rate was calculated.

**Result:**

Through propensity score matching, 43 pairs of cases were successfully matched. Compared with the S group, the D group had a shorter operation duration, ventilation duration, and ICU duration, as well as a lower drainage volume and total hospitalization cost. There was no significant difference between the two groups in the initial and highest serum lactate values after VSD closure, while the 6-h serum lactate value in the D group was significantly lower than that in the S group, and the 6-h serum lactate clearance rate in the D group was five times faster than that in the S group. In addition, the 6-h serum lactate clearance rate in the S group was mainly related to the operation time, CPB time, and ventilation time, while the 6-h serum lactate clearance rate in the D group was only related to the operation time.

**Conclusion:**

The initial and highest serum lactate levels were not significantly different between surgical repair and transthoracic device closure of VSD, but the 6-h serum lactate clearance rate of device closure was five times faster than that of surgical repair.

## Background

In the early stage of cardiac surgery, the overall oxygen consumption of the body increases significantly, while the oxygen supply often does not have a corresponding increase (1). In addition, many factors, such as anesthesia, cardiopulmonary bypass (CPB), and surgical operations, may result in tissue hypoxia and insufficient perfusion; thus, the body has an overall oxygen debt, and the balance of oxygen supply and demand is in a marginal state. As an intermediate product of anaerobic glycolysis, the level of serum lactate can indirectly reflect the tissue perfusion level, tissue oxygen supply, and peripheral circulation, and can be used as an indicator to reflect the oxygen supply and demand state of the body (2). Studies have suggested that the level of serum lactate could provide information about postoperative complications and poor prognosis (3). In addition, many articles have suggested that although CPB and hypothermia could reduce tissue metabolism and oxygen consumption, their non-physiological perfusion might lead to systemic and local perfusion insufficiency, leading to serum lactate accumulation (4, 5). Compared with surgical repair of ventricular septal defect (VSD), transthoracic device closure of VSD has the following advantages: it is less invasive, it requires no cardiopulmonary bypass, and it avoids hypothermia. Currently, there are few studies on whether tissue hypoperfusion occurs in the process of transthoracic device closure of VSD and whether there is a difference in metabolic cycles between these two treatments for VSD. Therefore, this study was conducted to compare the changes in serum lactate between these two treatments for VSD in pediatric patients.

## Materials and methods

### Patients

This study was a retrospective case analysis, and 314 pediatric patients with simple VSD were included in this study from October 2019 to October 2020. The inclusion criteria were as follows: (1) aged 5 years and below; (2) no other congenital heart disease; and (3) perimembranous VSD. The exclusion criteria were as follows: (1) congenital heart malformation other than VSD; (2) other systemic deformities; (3) severe pulmonary infection; (4) other surgical treatments performed before surgery; and (5) incomplete data. Preoperative routine examinations were completed for all cases, including chest radiography, transthoracic echocardiography, routine blood tests, biochemical tests, and inflammatory indicators. According to different surgical methods, the patients were divided into two groups: the surgical repair group (Group S, *n* = 212) and the transthoracic device closure group (Group D, *n* = 102).

### Surgical method

All patients were given intravenous inhalation combined with anesthesia. Median sternotomy, right submammary thoracotomy, and right vertical infra-axillary thoracotomy were used in the surgical repair of VSD, which also included CPB, cardiac arrest, and an autologous pericardial patch. Lower midline sternotomy was used in the transthoracic device closure of VSD, which included an occluder and a delivery system (6).

### Serum lactate value

The arterial lactate value was routinely measured at the end of surgery and ICU admission and then every 6 h until the patient was transferred from the ICU to a general ward. The initial serum lactate was defined as the serum lactate value measured when the patient was admitted to the ICU (7, 8). The formula for 6-h lactate clearance was as follows: 6-h lactate clearance = (the initial lactate-6 h postoperative lactate)/the initial lactate (9). In addition, depending on the length of ICU stay, we defined the “highest serum lactate” as the highest serum lactate value in the ICU. The “6-h serum lactate” was defined as the serum lactate value measured in the first 6 h. Serum lactate was measured as part of arterial blood gas analysis using a blood gas analyzer (GEM Premier 3000 blood gas analyzer; Instrument Laboratory, Bedford, MA).

### Statistical method

The statistical software used was SPSS statistics 26. If the measurement data conformed to a normal distribution after the normality test, a *t*-test was adopted; if not, a non-parametric test (Mann–Whitney Test) was adopted for comparative analysis. A chi-square test was used for counting data. To exclude differences in preoperative basic data, 1:1 propensity score matching (PSM) was used, and the caliper value was set to 0.2. Matched pair analysis was used after PS matching. Linear regression analysis and single-factor regression analysis were used for the correlation analysis. A *p*-value < 0.05 was considered statistically significant.

## Results

The basic data of the two groups of patients are shown in Table 1. There was no significant difference in the preoperative basic data except for sex and weight. Through propensity score matching, 43 pairs of cases were successfully matched. There was no statistical significance in the preoperative indices (*p* > 0.05).

**Table 1:** Comparison of basic data between surgery repair group (S group) and transthoracic device closure group (D group).

	**Before PSM**				**After PSM**			
	**S group** **(*****n** =* **212)**	**D group** **(*****n** =* **102)**	***P***	**SMD**	**S group** **(*****n** =* **43)**	**D group** **(*****n** =* **43)**	***P***	**SMD**
Gender (M/F)	116/106	54/58	0.490	0.1	20/23	22/21	0.415	0.17
Age (month)	18.1 ± 19.4	30.3 ± 18.7	0.028	0.02	22.3 ± 11.6	26.3 ± 10.7	0.205	0.06
Weight (kg)	8.32 ± 7.15	12.9 ± 8.8	0.096	0.12	9.72 ± 6.15	11.5 ± 4.2	0.223	0.09
Pulmonary pressure (mmHg)	40.4 ± 19.7	24.8 ± 12.4	0.03	0.15	36.4 ± 17.7	33.8 ± 10.4	0.168	0.02
VSD size (mm)	5.5 ± 2.1	3.7 ± 0.78	0.01	0.01	4.9 ± 1.1	4.2 ± 0.92	0.210	0.00

SMD, standard mean difference; PSM, propensity score matching.

All patients were successfully closed for VSD, and there was no reoperation, cerebrovascular accident, multiple organ dysfunction, or other serious postoperative complications (including complete atrioventricular block or malignant arrhythmia). A comparison of the perioperative data after matching between the two groups is shown in Table 2. There were significant differences in the operation time, duration of mechanical ventilation, length of ICU stay, drainage volume, and total cost between the two groups.

**Table 2:** Comparison of perioperative data between surgery repair group (S group) and transthoracic device closure group (D group) after PSM.

**Item**	**S group**	**D group**	***p***
Operation time (min)	124 ± 43	48 ± 14	0.012
Ventilation time (h)	23 ± 34	6 ± 5	0.000
ICU time (h)	35.5 ± 21.9	11.8 ± 7.8	0.041
Drainage (ml)	81.1 ± 45.0	52.2 ± 28.7	0.021
Total cost (ten thousand yuan)	4.6 ± 1.8	2.9 ± 1.3	0.000

PSM, propensity score matching.

The postoperative serum lactate value data of the two groups after matching are shown in Table 3. The *p*-values of the initial serum lactate value and the highest serum lactate value between the two groups were 0.317 and 0.643, respectively, showing no significant difference, while the *p*-values of the 6-h serum lactate value and 6-h serum lactate clearance rate were 0.029 and 0.001, respectively, showing a significant difference. In terms of the 6 h serum lactate clearance rate, the value of Group D was 0.25, indicating 25% serum lactate clearance at 6 h, while the value of Group S was −0.56, indicating that serum lactate was not cleared but increased by 56% at 6 h after surgery.

**Table 3:** Comparison of postoperative serum lactate between surgery repair group (S group) and transthoracic device closure group (D group) after PSM.

**Item**	**Group S**	**Group D**	***p***
First serum lactate (mmol/l)	1.68 ± 0.76	1.76 ± 0.67	0.317
6 h serum lactate (mmol/l)	2.50 ± 3.0	1.24 ± 0.90	0.029
The highest serum lactate (mmol/l)	2.97 ± 3.02	2.48 ± 1.02	0.643
6 h lactate clearance rate (%)	−0.56 ± 1.7	0.25 ± 0.48	0.001

PSM, propensity score matching.

To investigate the relationship between treatment methods and the 6-h serum lactate clearance rate, univariate regression analysis was used. The results of the data analysis after matching were as follows: *p* = 0.022, OR = 5.335, 95% CI: 1.267–22.463, which indicates a significant difference between the 6-h lactate clearance rates of the two groups, and the 6-h lactate clearance rate of Group D was 5.335 times higher than that of Group S.

To explore the related factors affecting the 6-h serum lactate clearance rate, regression analysis was performed, as shown in Tables 4, 5. In Group S, the 6-h serum lactate clearance rate after VSD repair in pediatric patients was mainly related to the operation time, CPB, and duration of mechanical ventilation, which were negatively correlated. In Group D, the 6-h lactate clearance rate was mainly related to the operation time and showed a negative correlation, indicating that the longer the operation time was, the slower the 6-h lactate clearance rate, and there was no significant correlation with age, weight, pulmonary artery pressure, VSD diameter, mechanical ventilation duration, and length of ICU stay.

**Table 4:** Regression analysis of 6h serum lactate clearance rate in the matched surgical repair (S group).

**Item**	**Regression coefficient**	**Standard error**	**Standard regression coefficient**	***t***	***P***	**95% CI**	
						**Lower**	**Upper**
Age	0.018	0.10	0.234	1.850	0.069	−0.001	0.038
Weight	0.029	0.034	0.112	0.112	0.389	−0.038	0.097
Pulmonary artery pressure	−0.023	0.010	−0.284	−2.273	0.27	−0.043	0.003
VSD size	−0.189	0.099	−0.243	−1.907	0.061	−0.387	0.009
Operation time	−0.013	0.002	−0.591	−5.625	0.000	−0.017	−0.008
CPB time	−0.025	0.004	−0.699	−6.264	0.000	−0.033	−0.017
Ventilation time	−0.001	0.000	−0.856	−12.70	0.000	−0.001	0.001
ICU time	0.000	0.000	0.080	0.601	0.551	0.000	0.000
Total cost	0.000	0.000	−0.079	−0.607	0.546	0.000	0.000

**Table 5:** Regression analysis of 6 h serum lactate clearance rate in the matched transthoracic device closure group (D group).

**Item**	**Regression coefficient**	**Standard error**	**Standard regression coefficient**	***t***	***P***	**95%CI**	
						**Lower**	**Upper**
Age	0.009	0.081	0.312	0.144	0.073	−0.042	0.025
Weight	0.019	0.124	0.193	0.210	0.253	−0.011	0.041
Pulmonary artery pressure	−0.001	0.010	−0.134	−1.733	0.321	−0.429	0.001
VSD size	−0.219	0.176	−0.059	−0.138	0.146	−0.188	0.013
Operation time	−0.048	0.051	−0.491	−7.625	0.017	−0.201	−0.080
Ventilation time	−0.018	0.001	−0.671	−1.704	0.204	−0.004	0.003
ICU time	0.000	0.000	0.011	1.410	0.281	0.000	0.000
Total cost	0.000	0.000	−0.109	−0.817	0.373	0.000	0.000

## Discussion

Pediatric patients who undergo surgical repair of VSD may develop tissue hypoxia due to CPB, ischemia–reperfusion injury, poor tissue perfusion, and cellular hypoxia (10–12). The change in blood lactate concentration can be used as a reliable indicator to reflect the oxygen supply and demand of the body and can be used to monitor and reflect tissue hypoxia. The normal range of serum lactate concentration in normal people is 1.0 ± 0.5 mmol/L. In postoperative patients, the normal serum lactate concentration is generally considered to be <2.0 mmol/L. In this study, the initial serum lactate concentration was <2 mmol/L. In a study by Ganushchak and his team, 109 cases underwent cardiac surgery with cardiopulmonary bypass, and the predictive sensitivity and specificity of the postoperative initial serum lactate concentration >5 mmol/L for complications were more than 70% and more than 80%, respectively (13). Other scholars concluded that serum lactate induced by CPB was significantly associated with patient prognosis, which further showed that the monitoring of arterial serum lactate after VSD closure had a certain clinical value in predicting the occurrence of complications and prognosis (14). Transthoracic device closure is another popular treatment for VSD in China (6). However, there are few studies on whether tissue hypoperfusion will also occur after transthoracic device closure of VSD. Therefore, the purpose of this study was to compare the changes in serum lactate between surgical repair and transthoracic device closure of VSD in pediatric patients.

The level of blood serum lactate can indirectly reflect the body tissue perfusion level, tissue oxygen supply, and peripheral circulation indices. Cheung et al. suggested that arterial serum lactate levels could be used as an early indicator for monitoring tissue hypoxia and could provide warning of postoperative complications and prognostic information for pediatric patients with congenital heart disease (3). According to the study from Noval-Padillo et al. although cardiopulmonary bypass and hypothermia reduced tissue metabolism and oxygen demand, nonphysiological perfusion still led to systemic or local hypoperfusion, leading to serum lactate accumulation (4).

In addition to direct observation of the serum lactate value, the lactate clearance rate can also effectively reflect the dynamic change in serum lactate level and has predictive value for the prognosis of severe pediatric patients after cardiac surgery (15). In this study, we found a negative 6-h lactate clearance rate in Group S, indicating that the lactate concentration continued to increase in the early postoperative period. Meanwhile, the 6-h lactate clearance rate reached 25% in Group D and was almost normal at 6 h. Nguyen and his colleagues suggested that a higher lactate clearance rate indicated improved organ function and an increased survival rate (16). This also suggested that continuous serum lactate elevation might be one of the warnings of postoperative complications, which is of great significance for the prevention of complications.

To further explore the related factors affecting the 6-h serum lactate clearance rate, we conducted a regression analysis. We found that surgical duration, mechanical ventilation duration, and cardiopulmonary bypass duration were significantly associated with 6-h lactate clearance in Group S. However, in Group D, the length of operation affected the 6-h serum lactate clearance rate, while the mechanical ventilation duration had no significant effect on the 6-h serum lactate clearance rate. We inferred that the main reason was that the mechanical ventilation duration was significantly shorter in Group D than in Group S. In most of the pediatric patients in this study who underwent transthoracic device closure of VSD, the endotracheal tube was removed within a few hours postoperatively. However, the exact reason still needs to be further explored. The 6-h serum lactate clearance rate of pediatric patients in both groups was negatively correlated with the length of operation, which might be due to the stress response caused by the trauma to the body during the operation.

## Limitation

This study had certain limitations. This study was a single-center retrospective study with a low sample size. Although we conducted PSM to minimize selectivity bias, the conclusion had certain limitations due to the reduction in sample size after matching, and it still needs to be further verified by a large sample randomized trial. Due to the use of retrospective case analysis in this study, limited by clinical habits, blood gas analysis was not routinely performed on the pediatric patients after they were transferred to the ward; thus, the serum lactate status of the pediatric patients during postoperative hospitalization could not be monitored.

## Conclusion

Although the surgical trauma of pediatric patients with VSD who underwent transthoracic device closure was less than that of surgical repair, the initial and highest serum lactate levels were not significantly different between the two groups, and the mean of the highest lactate level in the two groups was <3 mmol/l, but the 6-h serum lactate clearance rate of device closure was five times faster than that of surgical repair.

## Data availability statement

The original contributions presented in the study are included in the article/supplementary material, further inquiries can be directed to the corresponding authors.

## Author contributions

Y-RZ and J-SH conceived the idea. J-SH wrote the manuscript. Y-KC conducted the analyses. S-HL provided the data. HC and QC provided the technical and economic support. All authors contributed to the writing and revisions.
